# Electrostatics of Planar Multielectrode Sensors with Application to Surface Elastometry

**DOI:** 10.3390/s120911946

**Published:** 2012-08-29

**Authors:** Eugene Danicki, Yuriy Tasinkevych

**Affiliations:** Institute of Fundamental Technological Research of the Polish Academy of Sciences, 5B Pawinskiego str., 02-106 Warsaw, Poland; E-Mail: yurijtas@ippt.gov.pl

**Keywords:** interdigital transducer, surface elastometry, BIS-expansion

## Abstract

Systems of planar electrodes arranged on dielectric or piezoelectric layers are applied in numerous sensors and transducers. In this paper electrostatics of such electrode systems is presented and exploited in the analysis of distributed piezoelectric transducer dedicated to surface elastometry of biological tissues characterized by large Poisson modulus. The fundamental Matlab^®^ code for analyzing complex planar multiperiodic electrode systems is also presented.

## 1. Introduction

In many sensors, electric field is applied to investigate body by multiple electrodes which frequently can be considered periodic. This for example is the case of surface wave sensors of gas, actuators and linear ultrasonic motors utilizing planar metal strips as electrodes. Analysis of the field distribution and the electric property of electrode systems is usually necessary for the design and evaluation of the sensor parameters. The analysis of conducting strip is the general subject of this paper, and its application for elastometry is proposed as an example of its usefulness.

Typically, elastic properties can be evaluated by measuring the ultrasonic wave time of flight over certain distance. In the case of tissue however, small dimension of tissue sample and considerable wave damping makes the task difficult. In this paper we show how to measure the wave velocity within one ultrasonic transducer applying multiple strip electrodes on a piezoelectric layer applied to the tissue sample, in which case the detailed analysis of the strip system is indispensable because the frequency characteristic of the system, instead of the time of flight, is measured.

## 2. A Template Electric Field

The known identity [1] lays foundation for the presented analysis:

(1) $$\Gamma \left(\frac{1}{2}-\mu \right)\sum_{n=0}^{\infty }P_{n}^{\mu }(\cos\theta )\cos\left(n+\frac{1}{2}\right)v=\{\begin{array}{ll} \frac{\sqrt{\pi /2}\sin^{\mu }\theta }{{\left(\cos v-\cos\theta \right)}^{\mu +1/2}}, & 0\leq v<\theta ,\\ 0, & \theta <v<\pi , \end{array}$$

0 < *θ* < *π*, Re{*μ*} < 1/2. The involved Legendre function $P_{n}(\cdot )=P_{n}^{\mu }(\cdot )$ for *μ* = 0 has the following properties (for arbitrary real *ν* and *n* integer)

(2) $$\begin{array}{cc} P_{-\nu -1}=P_{\nu },\;P_{n}(-x)=S_{n}{\left(-1\right)}^{n}P_{n}(x), & S_{n}=\{\begin{array}{rr} 1, & n\geq 0,\\ -1, & n<0, \end{array} \end{array}$$

Two equations can be casted from the above identity which can be easily interpreted in electrostatic terms (the second equation results from the first one after substitutions *v* → *v* − *π* and *ϑ* → *ϑ* − *π*)

(3) $$\begin{array}{c} \sum_{n=-\infty }^{\infty }P_{n}(\Delta )e^{-j(n+1/2)Kx}=\{\begin{array}{ll} \frac{\sqrt{2}}{\sqrt{\cos\;\text{Kx}-\Delta }}, & |x|<w,\Delta =Kw,\\ 0, & w<|x|<\Lambda /2, \end{array}\\ \sum_{n=-\infty }^{\infty }S_{n}P_{n}(\Delta )e^{-j(n+1/2)Kx}=\{\begin{array}{ll} 0, & |x|<w,\\ \frac{-jS_{x}\sqrt{2}}{\sqrt{\Delta -\cos K_{x}}}, & w<|x|<\Lambda /2, \end{array} \end{array}$$

where the variable *ϑ* is replaced by *Kx* in order to accommodate the results to our purposes; Δ = cos *Kw*.

**Figure 1.**
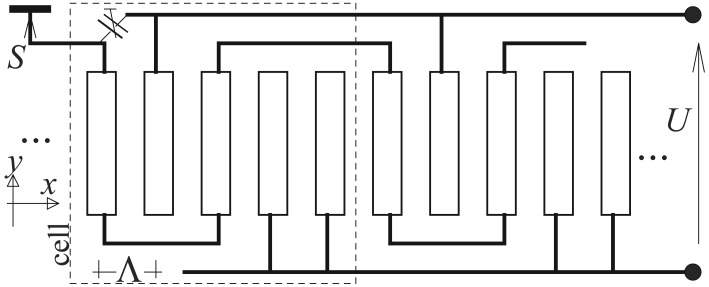
Periodic system of strips with external cross-less connections within the strip cells including five strips; the arrangement used in the discussed sensor.

The electrostatic interpretation of the above complementary set of functions is the following. The first equation is the Fourier expansion of the normal electric induction on planar periodic perfectly conducing strips arranged along axis *x* with period Λ = 2*π/K* (*K* is the corresponding “wave-number” of strips), and width 2*w*. As known from electrostatics, the electric charge on strips equals the normal induction discontinuity across the strips (*cf.* Figure 1) that is square-root singular at the strip edges. The second equation represent the Fourier expansion of the tangential electric field (similarly singular at the strip edges), naturally vanishing on the perfectly conducting strips (as opposite, the electric charge vanishes in the space between strips). These two expansions will be exploited as a template electric field in plane of planar system of periodic strips. Note that the Legendre functions involved in the left-hand side of the equations can be easily evaluated with help of efficient and robust numerical algorithm [2].

In order to make use of the above set of expansion, we need certain characterization of the electrostatic medium the strips to be embedded in. Consider the dielectric halfspace *z* > 0 of dielectric constant *ε*. Applying the electric potential *φ* = exp(−*jpx*) on the plane *z* = 0 (which axis is considered normal to the plane of strips), resulting in tangential electric field *E_x_* = −∇*_x_φ* = *jp* exp(−*jpx*), one easily evaluate that the excited normal electric induction (at *z* = 0+) satisfying the condition of vanishing field at *z* → ∞ is (superscript ‘+’ marks the considered field in the upper halfspace):

(4) $$D_{z}^{+}=-j\varepsilon S_{p}E_{x}^{+}$$

(neglecting the exponential terms), which is exactly the case of the corresponding harmonic components of the above two complementary Fourier series provided that the first equation is multiplied by *ε*. The corresponding field components are *P_n_*(Δ) exp(−*jnKx*) and *S_n_P_n_*(Δ) exp(−*jnKx*), for *D_z_* and *E_x_*, respectively. For the other halfspace *z* < 0 with dielectric constant *ε̄* and the field vanishing at *z* → −∞, the corresponding equation to the above one is $D_{z}^{-}=j\bar{\varepsilon }S_{p}E_{x}^{-}$, which allows us to evaluate the electric charge distribution on the plane *z* = 0 between these two halfspaces as $D=D_{z}^{+}-D_{z}^{-}=(1+\bar{\varepsilon }/\varepsilon )D_{z}^{+}$.

## 3. Arbitrary Potentials on Strips

The application of different electric potentials to subsequent strips breaks the electric field periodicity, hence the above Fourier expansion must be generalized into Bloch expansion of the planar electric field components by corresponding multiplication by *α_m_* exp(−*j*(*r* + *mK*)*x*) where *r* ∈ (0, *K*) is constrained to one Brillouin zone for uniqueness reason, yielding (*p_n_* = *r* + *nK* and series are simply rearranged; the superscript “+” is dropped, as well as subscript “*x*” at tangential field and *z* at normal induction):

(5) $$\begin{array}{c} D(x)=\sum_{n=-\infty }^{\infty }\sum_{m}{∊}_{e}\alpha_{m}P_{n-m}(\Delta )e^{-jp_{n}x},\\ E(x)=\sum_{n=-\infty }^{\infty }\sum_{m}jS_{n-m}\alpha_{m}P_{n-m}(\Delta )e^{-jp_{n}x}, \end{array}$$

(*α_m_* are arbitrary constants and *∊*_e_ = *ε* + *ε̄* is the surface effective dielectric permittivity). This multiplication does not change the support domains of the normal induction *D* (which remains *x* ∈ (−*w, w*) in the period Λ) nor *x* ∉ (−*w, w*) for the tangential field *E*.

Usually, the most interesting for applications are the strip potentials (which frequently are given) and the resulting strip charges or currents *J* = *jωD*, where *ω* is the angular frequency of the applied harmonic potentials to strips *V_l_* exp(*jωt*) (*l* is the particular strip number). In the considered case characterized by Equation (5), both these quantities can be evaluated explicitly using Dougall's identity [1], which, applying Equation (2), can be transformed into two equations:

(6) $$P_{-\nu }(-\Delta )=\frac{\sin\nu \pi }{\pi }\sum_{n}\frac{S_{n}P_{n}(\Delta )}{\nu +n},\;P_{-\nu }(\Delta )=\frac{\sin\nu \pi }{\pi }\sum_{n}\frac{{\left(-1\right)}^{n}P_{n}(\Delta )}{\nu +n}.$$

The *l*^th^ strip potential *V_l_* is evaluated by simple integration of electric tangential field, and, knowing that this potential is constant over the entire strip, the integral can be evaluated at centers of strips, that is at *x* = *l*Λ (*cf.* Figure 1); it is for given value of *r*:

(7) $$\begin{array}{c} V_{l}(r)=-\int E(x)dx\\ =-j\sum_{n}\alpha_{m}S_{n-m}P_{n-m}(\Delta )\frac{e^{-j(r+nK)x}{|}_{x=l\Lambda }}{-j(r+nK)}=\alpha_{m}V_{r}^{\left(m\right)}e^{-\text{jrl}\Lambda },\\ V_{r}^{\left(m\right)}=\frac{1}{K}\sum_{n\prime }\frac{S_{n^{\prime }}P_{n^{\prime }}(\Delta )}{n\prime +m+r/K}=\frac{\pi /K}{\sin\pi r/K}{\left(-1\right)}^{m}P_{-m-r/K}(-\Delta ). \end{array}$$

Analogous integral of the normal induction *D* has constant values between strips (where *D* = 0), which allows us to evaluate this integral at the center of space between strips (that is at *i*Λ − Λ/2 or at *i*Λ + Λ/2 on both side of *i*^th^ strip). The value of the *i*^th^ strip charge is the difference of the integral values at these two points: at the space centers after and before the *i*^th^ strip:

(8) $$\begin{array}{c} Q_{i}(r)=\int D\;dx{|}_{x=(i+1/2)\Lambda }-\int D\;dx{|}_{x=(i-1/2)\Lambda }=∊\alpha_{m}Q_{r}^{\left(m\right)}e^{-\text{jri}\Lambda },\\ Q_{r}^{\left(m\right)}=\int_{-\Lambda /2}^{\Lambda /2}\sum_{n}P_{n-m}(\Delta )e^{-j(r+nK)x}dx=\Lambda P_{-m-r/K}(\Delta ). \end{array}$$

Note that both *Q* and *V* are the spectral functions of *r* (moreover, *α_m_* may depend on *r* as well). They are, in fact, the Fourier transforms of discrete functions *Q_l_* and *V_l_* of values taken at the strip centers *x* = *l*Λ.

## 4. Strips on Layered Media

If strips are placed on a layered substrate, the Equation (4) is no longer valid as the value of *ε* depends on the component's wavenumber of the Bloch series, as it is shown in Appendix A for particular example of piezoelectric layer placed on a top of homogeneous elastic halfspace. The relation governing planar wavefield is:

(9) $$D(p)=-j∊(p)S_{p}E(p),\;∊(\left|p\right|>NK)\approx {∊}_{e}.$$

The fundamental feature of such system is that for large wavenumber value (say, for |*r* + *nK*| > *NK*, in certain acceptable approximation), the surface effective permittivity reaches its constant limit *∊_e_*, making Equation (4) valid for any *n* ∉ [− 1 − *N, N*] (assuming *r* > 0 within the allowed limits), that is for infinite number of Bloch components in the expansion Equation (5).

We apply the field expansion Equation (5) including summation over *m* with weight *α_m_*, which effectively means that each Bloch components of *D* and *E* is the sum like:

(10) $$\begin{array}{c} D_{r}(x)=\sum_{n=-\infty }^{\infty }D_{n}e^{-jp_{n}x},\;D_{n}={∊}_{e}\sum_{m}\alpha_{m}P_{n-m}(\Delta ),\\ E_{r}(x)=\sum_{n=-\infty }^{\infty }E_{n}e^{-jp_{n}x},\;E_{n}=\sum_{m}jS_{n-m}\alpha_{m}P_{n-m}(\Delta ), \end{array}$$

(*p_n_* = *r* + *nK*), where the appended subscript *r* marks the expansion dependence on spectral parameter *r* ∈ (0, *K*). The summation limits over *m*, depending on *N*, is usually quite small and can be established by careful inspection of the equations discussed below.

These equations result from Equation (9) applied for any pair of harmonic amplitudes (*D_n_, E_n_*) of the same harmonics exp(−*jp_n_x*) in the above expansion:

(11) $$\sum_{m}∊[1-S_{n-m}∊(p_{n})/{∊}_{e}]P_{n-m}(\Delta )\alpha_{m}=0,$$

for any *n* ∈ (−∞, ∞). Introducing *∊̄_n_* = *∊*(*p_n_*)/*∊_e_*, the expression in brackets becomes: 1 − *∊̄_n_S_n_*_−_*_m_* which is zero for |*n*| → ∞ provided that *m* is finite. Noticing that *∊̄_n_* = *S_n_* for *n* ∉ [−1 − *N, N*], according to Equation (9), it is easy to check that the discussed expression in bracket 1 − *S_n_S_n_*_−_*_m_* = 0 for any *n* < − 1 − *N* or *n* > *N* provided that − 1 − *N* ≤ *m* ≤ *N*. This yields the condition for acceptable domain of *m* in Equation (10), which effectively yields the proper truncation of the discussed infinite system of Equations [3]. It is evident that choosing larger *N* and wider range of *m*, according the above rule, will yield only trivial solution to the additionally included unknowns *α_m_*.

Note that the number of unknowns *α_m_* in Equation (10) is by one larger than the number of equations Equation (11), for |*n*| < *N*. The required additional equation results from the Kirchhoff's laws applied to the system of strips; the simplest one is the condition on the strip potentials exploiting Equation (7).

Evaluation of the strip voltages and currents (charges) requires integration of Equations (7, 8) over the spectral variable *r*, that is:

(12) $$\begin{array}{c} V_{l}=\frac{1}{K}\int_{0}^{K}\sum_{m}\alpha_{m}V_{r}^{\left(m\right)}e^{-\text{jrl}\Lambda }dr=V_{k}\delta_{kl},\\ I_{k}=j\omega Q_{k}=j\omega ∊\frac{1}{K}\int_{0}^{K}\sum_{m}\alpha_{m}Q_{r}^{\left(m\right)}e^{-\text{irk}\Lambda }dr, \end{array}$$

where *ω* = 2*πf* is the angular frequency of the applied voltage to strips (*f* is the frequency), *δ_lk_* is the Kronecker delta, *V_k_* are the given strip potentials, and *l*,*k* ∈ (−∞, ∞) are the strip numbers. It is evident from the first of the above equations that

(13) $$\sum_{m}\alpha_{m}(r)V_{r}^{\left(m\right)}=V_{l}e^{\text{jrl}\Lambda }.$$

This is the equation which, appended to Equation (11), allows one to evaluate all unknowns *α_m_* which, substituted into the second equation presented above, finally yields [4] the admittance relation for simple periodic strips:

(14) $$I_{k}=j\omega {∊}_{e}V_{l}\int_{0}^{K}R(r)e^{-jr(k-l)\Lambda }dr/K,$$

where *I_k_* is the current flowing to *k*th strip of unitary length, resulting from the *l*^th^ strip potential *V_l_*, and

$$R(r)=\frac{\sum_{m}\alpha_{m}P_{-m-r/K}(\Delta )}{\sum_{m}{\left(-1\right)}^{m}\alpha_{m}P_{-m-r/K}(-\Delta )}\sin\pi \frac{r}{K}.$$

## 5. Simple Elastometric Sensor

It is evident that the strip admittance depends on the piezoelectric and elastic property of the layered media on which the strips reside. In the case of biological tissue of large Poisson module (*ν* < 1/2), the most important and difficult task [5] is the determination of the shear wave velocity *v_t_* of the tissue, as the longitudinal wave velocity *v_l_* can be easily measured. The discussion below presents a method of evaluation of the shear wave wavenumber *k_t_* = *ω/v_t_* by means of strip admittance measured over certain frequency band.

In typical cases, the spectral function *R*(*r*) is singular at the wavenumber *r* = *k_R_* of Rayleigh wave (its value reduced to first Brillouin zone in the case of periodic system), but in order to make the measurements simpler in the considered case of elastometry, the layered system is chosen such that the Rayleigh–Lamb wave cannot propagate or it is not excited (see Appendix A for details). This feature allows one to perform simple numerical integration of Equation (14); below, the Fast Fourier transform is exploited for this task.

**Figure 2.**
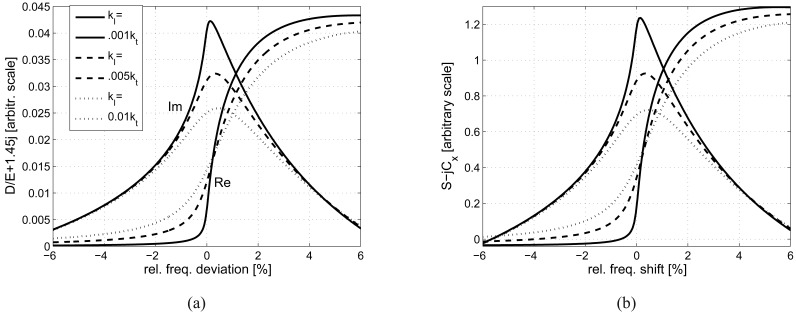
Typical frequency dependence (**a**) of normal induction excited on piezoelectric layer residing on a tissue-like body, and (**b**) of corresponding signal *S* in infinite periodic system.

The fundamental properties of the considered layered system are described by its effective dielectric permittivity *∊*(*r*; *ω*) for chosen value of *r* and variable *ω*, presented in Figure 2(a). This is the system response (in the complex amplitude of the normal induction) to the applied tangential electric wave-field on the system surface in the form exp *j*(*ωt* − *rx*). One can easily notice that the effective generation of shear waves propagating along the system surface takes place at frequency *f_o_* = *ω_o_*/(2*π*) of synchronism of the propagating wave with the delivered surface electric field spatial pattern: *ω_o_* = *rv_t_*. It should be noted that similar phenomenon of generation of subsurface longitudinal waves takes place for much lower frequency *rv_l_*. The proposed and analyzed measurements concern, in fact, evaluation of *∊*(*r*; *ω*) by means of the strip admittance dependence on angular frequency *ω*.

It is convenient to present first the results for infinite periodic system of 5-strip cells presented in Figure 1, where the alternate periodic potentials are applied to second, fourth and fifth strips (and periodically in other cells), and the excited current of the remaining strips makes the measured signal The system resemble a 3-phase interdigital transducer [6] and conveniently separates the generation and the signal circuits.

Due to the system exact periodicity and different voltages within periodic cells, the summation over the repeating strips (over indices *k, l*) in Equation (14) results in spectral “spikes” at *r* = *mK/M*, where *M* is the number of strips within cells, in the discussed case *M* = 5 and *m* = 0, 1, 2, 3, 4. The strip voltages and currents can thus be presented in the form (see Figure 1):

(15) $$\begin{array}{c} V_{l}=a_{m}e^{-j(mK/M)(l\Lambda )},\\ I_{k}=j\omega {∊}_{e}a_{m}R(mK/M)e^{-j(mK/M)(k\Lambda )}, \end{array}$$

where *l, k* = 0, 1, 2, 3, 4 and *a_m_* are coefficients to be evaluated from the circuit equations:

$$V_{0}=V_{2}=0,V_{1}-V_{3}=U,V_{4}=V_{3},\;I_{1}+I_{3}+I_{4}=0;$$

we intend to measure the combined current *I*_0_ + *I*_2_. Naturally, the measured current in real system would be the sum over all cells multiplied by the system aperture-width. For convenience of further discussion, we define the observed signal as *S* = (*I*_0_ + *I*_2_)/*ω* − *C*, where *C* is intended to reduce the signal resulting simply from the capacitance of the signal strips to the voltage-supplied strips (this reduction can be achieved by applying the trimmed capacitance to the supplied strip as shown in Figure 1).

In the case of finite number of cells, the Equation (14) must be applied with proper summation over the applied strip potentials and currents, within cells and over all cells in the sensor. Concerning strip potentials, the condition of equal but opposite bus-bar currents feeding the generating strips allows one to evaluate the voltage distributions: *V*^±^ of upper and lower bus-bars, respectively; *V*^+^ − *V*^−^ = *U* is the applied voltage to bus-bars. This corresponds to superposition of strip voltages *V*^±^ = ±*U*/2 and certain bias voltage *U^o^*. Noticing that *U^o^* would excite almost exclusively the thickness vibration of piezoelectric layer and normal longitudinal waves in the body, we may neglect *U^o^* yielding only certain signal bias like that caused by the strip mutual capacitances. Now, the above-mentioned double summation over strip cells results in multiplication of analytic *R*(*r*) in Equation (14) by

(16) $$\sum_{n=1}^{L}\sum_{m=1}^{L}e^{-jr(n-m)M\Lambda }={\left(L\sin\;x/x\right)}^{2},\;x=\pi \text{MLr}/K,$$

where *L* is the number of strip cells in the sensor.

**Figure 3.**
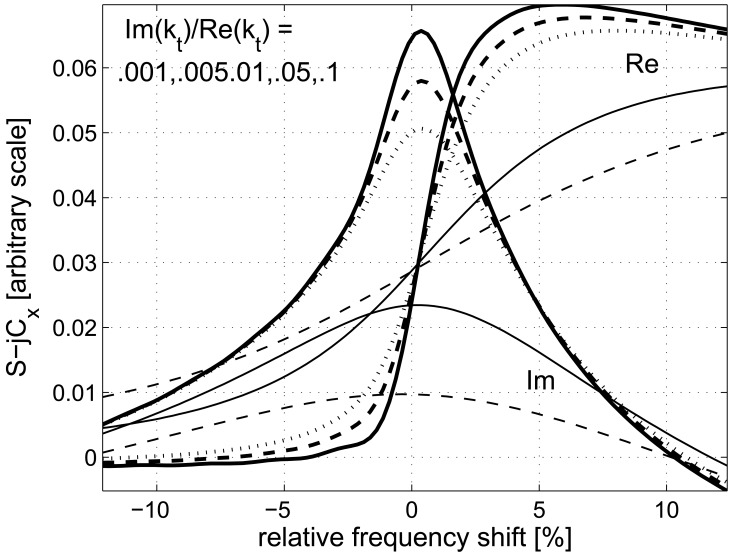
Example *S*(*f*) measured by sensor of 20 cells for several values of Im{*k_t_*}; the positions of max Im{*S*} are near zero frequency deviation in all cases.

The resulting signal *S*(*f*) as Figure 3 shows is somehow distorted in comparison with that shown in Figure 2(b), but still retains its useful features: max Im{*S*} appears at frequency close to *f_o_* (that is at zero frequency deviation in the figure) even for large Im{*k_t_*} yielding good estimation to Re{*k_t_*}, and the slope of Re{*S*} at *f_o_* dependence on Im{*k_t_*} can be used for estimation of the shear wave damping.

## 6. Multiperiodic Electrode System

In more general case, there are different strips within periodic cells. In the above-discussed sensor, for example, strips number 3 and 4 can be joined without any space between them. Such system is no longer simple-periodic; it is called [7] “multiperiodic” and the “template electric field” presented above must be suitably corrected. Appendix B presents the practical result of rather lengthy analysis [8,9] in form of a Matlab^®^ code for direct evaluation of the spatial Fourier expansion coefficients of the corrected template function $F_{n},n=0,1,2,3,\ldots (F_{-n}=F_{n}^{*})$ for periodic cells of period Λ. Each cells include even number *M* of strips and they are identically “wired” (by external connections) within all cells. This yields identical circuit equations except that the applied voltages can be arbitrarily scaled from cell to cell.

The electric fields is constructed using these Fourier coefficients analogously to Equations (5) and (10):

(17) $$\begin{array}{c} D(x)=\sum_{n=-\infty }^{\infty }\sum_{m}{∊}_{e}\alpha_{m}F_{n-m}e^{-j(r+nK)x},\\ E(x)=\sum_{n=-\infty }^{\infty }\sum_{m}jS_{n-m}\alpha_{m}F_{n-m}e^{-j(r+nK)x}, \end{array}$$

where spectral variable *r* ∈ (0, *K*), *K* = 2*π*/Λ. There is certain difficulty in numerical evaluation of potentials and charges of strips resulting from the above equation. Namely, integration of *D*(*x*) and *E*(*x*) may yield infinity if *r* = 0 (which happens at *i* = 0).

This can be avoided well by applying small *r* instead of 0 when applying the FFT procedure for integration (*r* ∼ 10^−4^ is a good choice). Taking the output of FFT{[*jS_n_*_−_*_m_F_n_*_−_*_m_*/(*r* + *nK*)]} at the given strip center yields the sought potential of the strips in the cell, and taking the difference of FFT{[*jF_n_*_−_*_m_*/(*r* + *nK*)]} at the space centers after and before the strip yields the given strip charge, $V_{l}^{\left(m\right)}$ and $Q_{l}^{\left(m\right)}$ respectively, *l* = 0, …, *M* (expressions in brackets are the input vectors of FFT algorithm). These values are exploited in the circuit equations for strip currents and voltages in order to evaluate *α_m_*, other necessary equations result from Equation (4) (put *p* = *r* + *nK*) resulting in Equation (11) where *P_n_*_−_*_m_* should be replaced by *F_n_*_−_*_m_*.

## 7. Conclusions

Modern electronic technology allows one to easily fabricate planar system of strips, which contributes to their wide applications in many electronic devices including sensors (SAW gas sensors, for instance) and actuators (piezoelectric linear motors, for instance). Rigorous electric field analysis is usually necessary for the design and evaluation of electric properties of such devices. In this paper, a method of analysis of periodic strips or periodic groups of strips (with arbitrary width and spacing within cells) is presented in interesting application for surface elastographic sensor, which may contribute to better accuracy of measurements of tissue properties required in medical investigation and diagnosis of skin [10] for instance.

## Appendix A

Consider an isotropic elastic halfspace *z* < 0 characterized by Lamè constants *λ, μ* and mass density *ρ*, resulting in acoustic wave velocities $v_{l}=\sqrt{(2\mu +\lambda )/\rho }$ and $v_{t}=\sqrt{\mu /\rho }$, respectively for longitudinal and shear waves. The plane acoustic wave-fields include displacements *u*_1,2_ and normal traction *T*_3_*_i_, i* = 1, 3. The harmonic wave-field is considered in the form: exp(*jωt* − *jpx* − *jq_i_z*) where $q_{i}=\sqrt{{\left(\omega /v_{i}\right)}^{2}-p^{2}},i=l,t$ with sign chosen to satisfy the radiation condition [4] at *z* → ∞; *p* is arbitrary. The wave-field is the superposition of longitudinal and shear wave-components (we drop the time-dependence):

(18) $$\begin{array}{c} \left[\begin{array}{c} u_{1}\\ u_{3} \end{array}\right]=\left[\begin{array}{cc} p & -q_{t}\\ q_{l} & p \end{array}\right]\;\left[\begin{array}{cc} e^{-jq_{l}z} & 0\\ 0 & e^{-jq_{t}z} \end{array}\right]\;\left[\begin{array}{c} F_{l}\\ F_{t} \end{array}\right]\;e^{-\text{jpx}},\\ \left[\begin{array}{c} T_{31}\\ T_{33} \end{array}\right]=\left[\begin{array}{c} \mu u_{3,1}+\mu u_{1,3}\\ \lambda u_{1,1}+(\lambda +2\mu )u_{3,3} \end{array}\right]\;,f_{,i}=\partial f/\partial x_{i} \end{array}$$

(*x*_1_ = *x, x*_3_ = *z*). For sliding contact with the piezoelectric layer at *z* = 0, one easily obtains the *p*-dependent function evaluated at *z* = 0:

(19) $$Z=T_{33}/u_{3}.$$

Much more complicated is the characterization [4] of piezoelectric layer; the theory yields the dependence of electric tangential field (*E* = *jpφ*) and normal induction (*D* = *D*_3_) on the layer upper side, which is considered traction-free on normal displacement (*u*_3_) and traction *T*_33_ applied to its bottom side which is considered metalized:

(20) $$-jp\left[\begin{array}{c} u_{3}\\ \varphi \end{array}\right]=\left[\begin{array}{cc} Z_{11} & Z_{12}\\ Z_{21} & Z_{22} \end{array}\right]\;\left[\begin{array}{c} T_{33}\\ D_{3} \end{array}\right].$$

Substitution of Equation (19) yields Equation (9).

## Appendix B

Consider even number *M* of strips placed within the domain [0, 1]: the strip left and right edges are specified in two-column matrix *g* of *M* rows (for the algorithm convenience the edge positions are modified in the line two). The algorithm presented below yields the data vector *F* = [*F_n_*], for specified numbers of harmonics: *n* = 0, …, *N* − 1.

ne=length(g);	%ne even!
g=g-sum(sum(g′)′/2)/ne;	%shifting
h=1i*pi*sum(g′)′;	
y= [1i;zeros(N-1,1)];	
for m=1:ne;	
t=cos(pi*(g(m,2)-g(m,1)));	
a=1i;	
b=1i*t;	
z=[zeros(1,N-2) b*exp(h(m)) a];	
for l=2:N-1;	
c=(t*(2*l-1)*b-(l-1)*a)/l;	
a=b;	
b=C;	
z(N-l)=c*exp(l*h(m));	
end;	
for i=N:-1:1;	
y(i)=2*z(1+N-i:N)*y(1:i);	
end;	
end;	
y=[zeros(ne/2,1);y];	
F=y(1:N);	
